# Therapeutic Potential of Autophagy in Glioblastoma Treatment With Phosphoinositide 3-Kinase/Protein Kinase B/Mammalian Target of Rapamycin Signaling Pathway Inhibitors

**DOI:** 10.3389/fonc.2020.572904

**Published:** 2020-10-02

**Authors:** Qin Xia, Mengchuan Xu, Pei Zhang, Liqun Liu, Xinyi Meng, Lei Dong

**Affiliations:** School of Life Science, Beijing Institute of Technology, Beijing, China

**Keywords:** glioblastoma, autophagy, PI3K/AKT/mTOR inhibitors, drug resistance, combination therapy

## Abstract

Glioblastoma (GB) is the most malignant and aggressive form of brain tumor, characterized by frequent hyperactivation of the phosphoinositide 3-kinase (PI3K)/protein kinase B (AKT)/mammalian target of rapamycin (mTOR) signaling pathway. PI3K/AKT/mTOR inhibitors have a promising clinical efficacy theoretically. However, strong drug resistance is developed in GB against the PI3K/AKT/mTOR inhibitors due to the cytoprotective effect and the adaptive response of autophagy during the treatment of GB. Activation of autophagy by the PI3K/AKT/mTOR inhibitors not only enhances treatment sensitivity but also leads to cell survival when drug resistance develops in cancer cells. In this review, we analyze how to increase the antitumor effect of the PI3K/AKT/mTOR inhibitors in GB treatment, which is achieved by various mechanisms, among which targeting autophagy is an important mechanism. We review the dual role of autophagy in both GB therapy and resistance against inhibitors of the PI3K/AKT/mTOR signaling pathway, and further discuss the possibility of using combinations of autophagy and PI3K/AKT/mTOR inhibitors to improve the treatment efficacy for GB. Finally, we provide new perspectives for targeting autophagy in GB therapy.

## Introduction

Glioblastoma (GB), a World Health Organization (WHO) grade IV glioma, is the most common and malignant primary brain tumor in adults, accounting for the majority of gliomas and representing 45.2% of malignant gliomas (1, 2). GB is characterized by nuclear atypia, cell polymorphism, and rapid proliferation, and patients with GB have a median survival of <15 months (3). Currently, the standard treatment for GB is surgical resection followed by radiotherapy and tumor adjuvant chemotherapy with the methylated agent temozolomide (TMZ) drug. However, due to the development of chemotherapy resistance, standard treatment only improves the median survival from 12 to 14.6 months (4). Thus, chemotherapy resistance is one of the most important reasons for GB treatment failure and high recurrence (5).

Several studies have shown that the abnormal hyperactivation of the phosphoinositide 3-kinase (PI3K)/protein kinase B (AKT)/mammalian target of rapamycin (mTOR) pathway caused by gene mutations is estimated to be around 88% in GB (6), such as those leading to overexpression of epidermal growth factor receptor (EGFR), activation of phosphatidylinositol-4,5-bisphosphate 3-kinase catalytic subunit alpha (PIK3CA) or phosphoinositide-3-kinase regulatory subunit 1 (PIK3R1), or loss of phosphatase and tensin homolog (PTEN) expression, which contributes to cell survival and the development of drug resistance, leading to reduced therapeutic effect of TMZ (6, 7). Therefore, inhibition of the PI3K/AKT/mTOR signaling pathway is an ideal strategy to improve the treatment of GB. Many studies have demonstrated that autophagy is critically involved in the treatment of GB with inhibitors of the PI3K/AKT/mTOR signaling pathway (8–10). Autophagy is an evolutionarily conserved and regulated complex process of degradation and recycling of cellular components participating in organelle turnover (11). Autophagy has been reported to affect the cell growth, migration, and invasion abilities of GB cells (12, 13). The PI3K/AKT/mTOR pathway is one of the most important metabolic pathways, which mediate the inhibition of autophagy by negatively regulating the activity of autophagy-related proteins, and is involved in cell growth. In this case, the induction of autophagy by the PI3K/AKT/mTOR inhibitors may suppress the growth of cancer cells. However, autophagy has a dual role in tumor cells as pro-survival and pro-death mechanisms (14). Accumulating evidence has revealed that autophagy can protect cells against the effects of the PI3K/AKT/mTOR inhibitors, leading to the development of drug resistance (15). Accordingly, autophagy can also play a dual role in the treatment of GB with the PI3K/AKT/mTOR signaling pathway inhibitors, and understanding these effects is necessary for improving the treatment of GB.

This review summarizes the dual role of autophagy in the treatment of GB with inhibitors of the PI3K/AKT/mTOR pathway. On the one hand, the inhibitors induce autophagy to play an antitumor effect. However, along with the development of drug resistance, autophagy prevents tumor cells from cell death; in this case, a combination of autophagy inhibitors should be encouraged. In addition, we discuss when the combination of autophagy inhibitors is more effective. Since the main focus of this review is the therapeutic potential role of autophagy in the treatment of GB, we also review the current GB standard treatment and provide evidence that targeting autophagy improves the efficacy of chemotherapy and radiotherapy.

## Rationale of Targeted Phosphoinositide 3-Kinase/Protein Kinase B/Mammalian Target of Rapamycin Signaling Pathway in Glioblastoma

### Hyperactivation of the Phosphoinositide 3-Kinase/Protein Kinase B/Mammalian Target of Rapamycin Pathway

**Figure 1:**
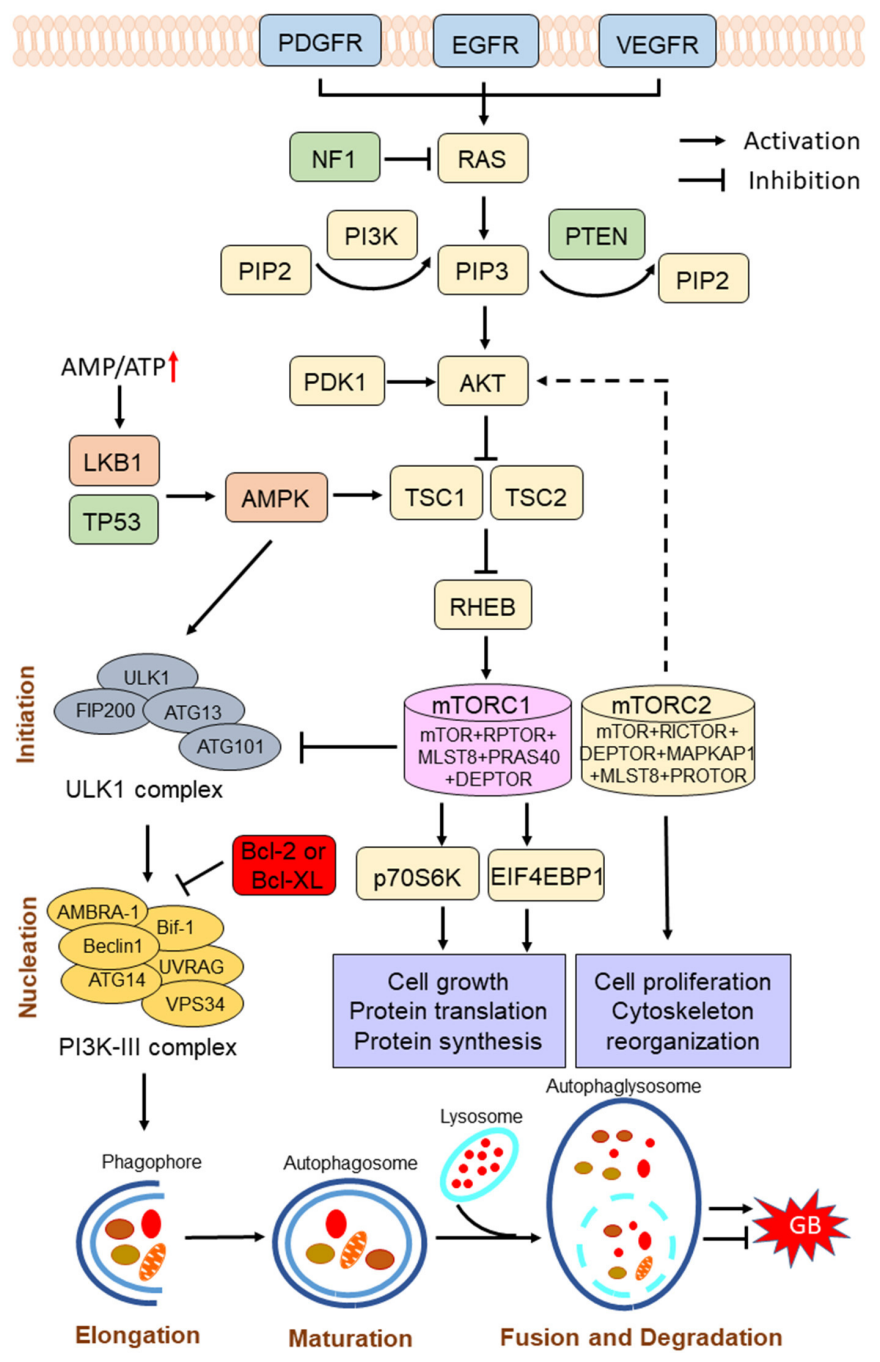
Scheme of the phosphoinositide 3-kinase (PI3K)/protein kinase B (AKT)/mammalian target of rapamycin (mTOR) pathway in the regulation of autophagy process. The PI3K/AKT/mTOR signaling pathway is activated by various receptor tyrosine kinases (RTKs), such as epidermal growth factor receptor (EGFR), vascular endothelial growth factor receptor (VEGFR), and platelet-derived growth factor receptor (PDGFR), while the pathway is negatively regulated by phosphatase and tensin homolog (PTEN). Hyperactivation of the PI3K/AKT/mTOR pathway inhibits autophagy, and suppression of the PI3K/AKT/mTOR pathway induces autophagy. mTOR complex I (mTORC1) plays a key role in the regulation of autophagy process. Under conditions of nutrient sufficiency, mTORC1 is activated and inhibits autophagy. Under starvation or low energy, AMP-activated protein kinase (AMPK) is activated and mTORC1 is inactivated, leading to the initiation of autophagy. Induction of autophagy can be summarized into several phases: initiation, nucleation, elongation, maturation, fusion, and degradation. Autophagy has a dual role in pro-survival or pro-cell death in GB progression. NF1, neurofibromin 1; PIP2, phosphatidylinositol-4,5-bisphosphate; PIP3, phosphatidylinositol-3,4,5-trisphosphate; PDK1, 3-phosphoinositide-dependent kinase-1; TSC1/2, tuberous sclerosis complex 1/2; RHEB, Ras homolog enriched in brain; LKB1, liver kinase B1; p70S6K, 70-kDa ribosomal protein S6 kinase; EIF4EBP1, eukaryotic translation initiation factor 4E-binding protein 1.

The phosphoinositide 3-kinase (PI3K)/protein kinase B (AKT)/mammalian target of rapamycin (mTOR) pathway is activated by various receptor tyrosine kinases (RTKs), such as EGFR, which binds the adaptor protein growth factor receptor-bound protein 2 (GRB2) and GRB2-associated binding protein 1 (GAB1) to recruit and activate PI3K at the cell membrane (16). Activated PI3K converts phosphatidylinositol-4,5-bisphosphate (PIP2) into the second messenger phosphatidylinositol-3,4,5-trisphosphate (PIP3) (17). Once formed, PIP3 recruits 3-phosphoinositide-dependent kinase-1 (PDK1) and promotes the activation of AKT in the plasma membrane. Activated AKT subsequently phosphorylates the tuberous sclerosis complex 2 (TSC2), thereby preventing the formation of the TSC2/TSC1 complex, which acts as a GTPase for Ras homolog enriched in brain (RHEB), a GTP-binding protein that activates mTOR complex I (mTORC1) (18) (Figure 1). mTOR, an evolutionarily conserved protein kinase, plays a key role at the intersection of the pathway that coordinately regulates the balance between cell growth and autophagy in response to nutritional status, growth factors, and stress signals (19). mTOR forms two distinct protein complexes, namely, the rapamycin-sensitive mTORC1, and the rapamycin-insensitive mTORC2. The mTORC1 consists of mTOR, regulatory-associated protein of mTOR complex 1 (RPTOR), mTOR-associated protein, LST8 homolog (MLST8), proline-rich AKT substrate of 40-kDa (PRAS40), and DEP domain-containing mTOR-interacting protein (DEPTOR). The 70-kDa ribosomal protein S6 kinase (p70S6K) and the eukaryotic translation initiation factor 4E-binding protein 1 (EIF4EBP1) are essential downstream proteins of mTORC1 (20). The mTORC1 promotes cell growth by phosphorylating p70S6K to induce protein synthesis and phosphorylating EIF4EBP1 to initiate translation (21). The mTORC2 consists of mTOR, RPTOR-independent companion of mTOR complex 2 (RICTOR), MLST8, MAPK-associated protein 1 (MAPKAP1), DEPTOR, and protein observed with RICTOR (PROTOR) (22). The mTORC2 phosphorylates protein kinase C (PKC)δ, PKCζ, PCKγ, and PKCε, as well as AKT at Ser473 to regulate cell proliferation and cytoskeleton reorganization (10, 22). Notably, the PI3K/AKT/mTOR is the only negatively regulated signaling pathway of PTEN. PTEN decreases the PIP3 levels through blocking the phosphorylation process of PIP2 to PIP3 and suppresses the activation of the PI3K/AKT/mTOR signaling pathway (23).

In addition, the most common genetic aberrations in GB, including mutations in *EGFR*, neurofibromin 1(*NF1*), *PIK3CA, PIK3R1, PTEN*, and *TP53*, promote the hyperactivation of the PI3K/AKT/mTOR pathway, and lead to GB progression and relapse during drug treatment. In depth, phenotypically, the overexpression or amplifications of *EGFR* and the expression of the EGFRvIII variant (*EGFR* lacking exons 2–7 encoding the extracellular domain) are commonly observed in glioma and are associated with resistance, leading to cell proliferation, differentiation, and survival through the activation of the PI3K/AKT/mTOR pathway (16, 24). Additionally, gene mutation or deletion of *NF1*, a negative regulator of the RAS signaling pathway, occurs in 15–18% of primary GBs, leading to activation of the downstream PI3K/AKT/mTOR pathway (23). Furthermore, deregulating mutations of the catalytic (PIK3CA) or regulatory (PIK3R1) domains of PI3K, which are also observed in GB, induce the activity of these enzymes to promote the PI3K/AKT/mTOR pathway (23). *PTEN* loss of function is frequently observed in GB, including mutations within the *PTEN* coding sequence, deletion of the second allele, and impairment of the catalytic activity of PTEN (18). *PTEN* loss leads to increased activation of the downstream mTOR, thereby inhibiting autophagy (25). *TP53* is one of the most frequently deregulated genes in GB; its encoded protein p53 binds to the β 1, β 2, and γ subunits of AMP-activated protein kinase (AMPK) and PTEN promoter to activate their expression, which can affect the activity of the PI3K/AKT/mTOR signaling pathway (26).

### Phosphoinositide 3-Kinase/Protein Kinase B/Mammalian Target of Rapamycin Pathway Inhibitors in Glioblastoma Treatment

Based on the poor outcome of the hyperactivation of the PI3K/AKT/mTOR signaling pathway, the inhibition of this signaling pathway is an ideal strategy for the treatment of GB. However, GB cells develop strong resistance to PI3K/AKT/mTOR inhibitors during its treatment. This review introduces the main inhibitors of the PI3K/AKT/mTOR pathway (Table 1) and focuses on the drug resistance mechanisms.

**Table 1:** Therapeutic agents regulate phosphoinositide 3-kinase (PI3K)/protein kinase B (AKT)/mammalian target of rapamycin (mTOR) pathway or autophagy for glioblastoma (GB) treatment.

**Compound**	**Target**	**Effect**	**References**
Gefitinib	Epidermal growth factor receptor (EGFR) inhibitor	Enhanced antitumor effect	(10)
Erlotinib	EGFR inhibitor	Enhanced antitumor effect	(10)
BYL719	PI3K inhibitor	Enhanced antitumor effect	(27)
GS-1101	PI3K inhibitor	Enhanced antitumor effect	(27)
BKM120	PI3K inhibitor	Enhanced antitumor effect	(28, 30)
PX-866	PI3K inhibitor	Enhanced antitumor effect	(29, 80)
GDC-0941	PI3K inhibitor	Enhanced antitumor effect	(9)
3MA	PIK3C3 inhibitor	Enhanced antitumor effect	(96)
Wortmannin	PIK3C3 inhibitor	Enhanced antitumor effect	(37, 96)
LY294002	PIK3C3 inhibitor	Enhanced antitumor effect	(37, 96)
Perifosine	AKT inhibitor	Enhanced antitumor effect	(31)
Triciribine/API-2	AKT inhibitor	Enhanced antitumor effect	(32)
Rapamycin	mTORC1 inhibitor	Enhanced antitumor effect	(10)
Everolimus	mTORC1 inhibitor	Enhanced antitumor effect	(33, 87)
Torin1	mTORC1/mTORC2 inhibitor	Enhanced antitumor effect	(22)
PP242	mTORC1/mTORC2 inhibitor	Enhanced antitumor effect	(22, 71)
CC214-1/2	mTORC1/mTORC2 inhibitor	Enhanced antitumor effect	(22)
AZD8055	mTORC1/mTORC2 inhibitor	Enhanced antitumor effect	(22)
OSI-027	mTORC1/mTORC2 inhibitor	Enhanced antitumor effect	(22)
Curcumin	Polyphenol	Induced cell death	(8)
NVP-BEZ235	PI3K/mTOR inhibitor	Enhanced antitumor effect	(34)
XL765	PI3K/mTOR inhibitor	Enhanced antitumor effect	(35)
PI-103	PI3K/mTOR inhibitor	Enhanced antitumor effect	(81)
CMG002	PI3K/mTOR inhibitor	Enhanced antitumor effect	(85)
Chloroquine(CQ)	Lysosomal pH	Enhanced radiation sensitization	(94)
Hydroxychloroquine (HCQ)	Lysosomal pH	Enhanced radiation sensitization	(94)
Bafilomycin A1	Vacuolar ATPase	Enhanced antitumor effect	(95)
SBI-0206965	ULK1 inhibitor	Enhanced antitumor effect	(86)
SAR405	VPS18/PIK3C3 inhibitor	Reduced proliferation	(87)
Lys05	Unknown	Decreased cell growth	(97)

Tyrosine kinase inhibitors (TKIs) specifically inhibit RTKs by binding the tyrosine kinase domain, which prevents RTK phosphorylation and activation of signaling cascades, such as the PI3K/AKT/mTOR pathway. Clinically, the most widely used anti-EGFR inhibitors to treat recurrent GB are gefitinib and erlotinib (10). However, the EGFR T790M mutation, acquired RTK MET gene amplification and PIK3CA mutations, share the same effect of reestablishing the downstream PI3K/AKT/mTOR pathway in the presence of the anti-EGFR inhibitors, ultimately converting a TKI-sensitive tumor into a TKI-resistant tumor (27).

The first-generation anti-PI3K inhibitors are BYL719 (alpelisib) and GS-1101 (idelalisib/CAL-101), which specifically inhibit PIK3CA and phosphatidylinositol-4,5-bisphosphate 3-kinase catalytic subunit delta (PIK3CD), respectively (27). Although these anti-PI3K inhibitors have effective therapeutic effects, their poor solubility and high toxicity have limited their clinical application. Currently, a new generation of pan-PI3K inhibitors include BKM120 (buparlisib) and PX-866 (sonolisib), which have high stability and low side effects (28, 29). Some studies have demonstrated that the treatment with BKM120 also inhibits AKT phosphorylation, leading to the inhibition of glioma cell proliferation (30). In addition, *in vivo* studies have shown that BKM120 reduces the volumetric increase of the tumor and prolongs survival of nude rats harboring human GB xenografts (30).

One of the most promising AKT inhibitors is perifosine, a lipid-based inhibitor, which inhibits AKT activity by preventing the transport of AKT to the cell membrane. However, the use of perifosine monotherapy in solid tumors has been disappointing due to gastrointestinal and constitutional toxicities (31). Given its limited efficacy, an alternative strategy of a combination of perifosine with other targeted agents should be pursued to enhance cytotoxicity and overcome chemotherapeutic resistance through inhibition of the AKT pathway. Triciribine/API-2, another AKT (all three family members of AKT, including AKT1, AKT2, and AKT3) inhibitor, inhibits AKT2 phosphorylation at both sites (Thr309 and Ser474) and EGF-induced phosphorylation of all three AKT isoforms (32). However, triciribine at high doses has many serious toxicities, and for this reason, it has not been used in combination with standard chemotherapy or radiotherapy in clinical trials.

Inhibition of mTOR induces autophagy by promoting the activation of several autophagy-related proteins involved in the initial stage of membrane isolation. Rapamycin, an early stage inhibitor of mTOR, binds the 12-kDa FK506-binding protein (FKBP12), which stabilizes the RPTOR–mTOR interaction and blocks the mTOR phosphorylation activity, ultimately leading to induction of autophagy (10). Rapamycin also mediates cell death after inhibition of mTOR in both GB cells with wild-type *PTEN* gene and GB cells without *PTEN* gene, indicating that a variety of signaling pathways are involved in autophagy regulation (14). Everolimus, a derivative of rapamycin and known as rapalogs, specifically inhibits the mTORC1 signaling pathway (33). However, rapamycin and its analogs (rapalogs) have limited clinical use due to their lack of activity against mTORC2, leading to incomplete inhibition of mTORC1 downstream targets, which eventually induces PI3K reactivation (22). Recent evidence reveals that dual blockade of mTORC1 and mTORC2 may potentially overcome the limitations of mTORC1 inhibitors and provide an effective therapeutic strategy for patients with GB (22). Recently, a new generation of ATP-competitive mTOR kinases that irreversibly block both mTORC1 and mTORC2 activation have been developed, including Torin1, PP242, CC214-1/2, AZD8055, and OSI-027 (22).

Some studies have shown that continuous inhibition of mTOR leads to reactivation of AKT *via* negative feedback inhibition and targeting upstream PI3Ks (PIK3CA, B, G, D) rather than AKT or mTOR, which is thought to be responsible for the achievement of the higher impact (27). Thus, targeting PI3Ks in the PI3K/AKT/mTOR pathway may represent a promising therapy. However, clinical trials with PI3K inhibitors alone in monotherapy have failed to achieve significantly successful outcomes. Currently, a multitargeted strategy combining PI3K inhibitors with other targeted drugs is a successful strategy. For instance, targeting both PI3K and mTOR with NVP-BEZ235, a dual PI3K/mTOR inhibitor, resulted in an antiproliferative and antitumoral activity in cancer cells (34). The combination of XL765, another dual PI3K/mTOR inhibitor, with TMZ had better curative effects both *in vivo* and *in vitro* (35). According to these findings, future perspectives for GB therapy should focus on the multitargeted strategy.

## The Role of Autophagy in Glioblastoma Therapy With the Phosphoinositide 3-Kinase/Protein Kinase B/Mammalian Target of Rapamycin Pathway Inhibitors

At present, autophagy research is in a logarithmic growth phase, driven by a new understanding of the highly important roles of autophagy in health and diseases, including cancer. Autophagy is thought to have both pro-survival and pro-death roles in tumor cells (14). Furthermore, autophagy is an important mechanism that can be regulated by the PI3K/AKT/mTOR pathway. Accordingly, based on the role of autophagy in GB, the induction of autophagy by the PI3K/AKT/mTOR inhibitors can also have a dual role in GB therapy. Understanding these effects is critical for the study of drug resistance and development of an effective therapy for GB.

### Dual Role of Autophagy in Glioblastoma

Autophagy has dual cytoprotective and pro-cell death roles in GB tumorigenesis. On the one hand, autophagy provides necessary nutrition and energy to promote tumor survival under physiological or pathological conditions, such as nutrient deficiency, hypoxia, ER stress, protein misfolding and aggregation, and metabolic stress, etc. (36, 37). Additionally, therapeutic agents, such as radiotherapy and chemotherapy, overinduce protective autophagy in cancer cells, leading to cell desensitization and eventually development of resistance to therapy. On the other hand, autophagy suppresses tumor growth by degrading tumorigenic proteins and oxidized products that contribute to tumor progression (38, 39). Accordingly, understanding the detailed dual mechanism of autophagy and how to distinguish it in different conditions is crucial to improve anticancer drug efficacy.

### Autophagy as a Tumor Suppressor

Low expression or deletion of genes related to autophagy initiation and elongation have been detected in GB, such as unc-51-like autophagy-activating kinase 1/2 (*ULK1/2)*, FAK family kinase-interacting protein of 200 kDa (*FIP200)*, Beclin 1 (*BECN1)*, UV radiation resistance associated (*UVRAG)*, autophagy-related 4C cysteine peptidase (*ATG4C*), and autophagy-related 5 (*ATG5)*, suggesting that autophagy plays a suppressive role in GB tumorigenesis (40, 41). Mechanically, autophagy inhibits GB progression through various manners. The first mechanism is inducing GB cell senescence, thereby permanently blocking the cell cycle and strongly inhibiting tumorigenesis. For instance, activation of autophagy by inhibiting the mTOR signaling was reported to increase TMZ-induced cell senescence (42). Induction of autophagy by starvation or pharmacological inhibition of the mTOR complexes has been also shown to impair GB cell migration and invasion (43). Second, autophagy also induces cell death caused by inhibited necrosis through limiting the inflammatory response, which favors necrosis-associated tumor cell growth (44). In addition, chemoradiotherapy for GB usually leads to apoptotic death, but recent research shows that chemoradiotherapy also causes autophagy-dependent cell death, and this process depends on a specific autophagy activation mechanism (45). The third mechanism of autophagy-mediated tumor suppression is through induction of apoptosis *via* ATG proteins. For example, ATG5 regulates the link of autophagy and apoptosis. A study by Guk Heui et al. found that silencing *ATG5* in U87, U373, and LN229 glioma cell lines effectively reduced radiation-induced autophagy, which significantly decreased apoptosis and contributed to cell survival (46). The fourth and last mechanism is the autophagy-mediated regulation of stem cell maintenance. Self-renewal and differentiation of stem cells into several types of cells are important processes in carcinogenesis, as well as in development and tissue renewal. Several studies indicate that autophagy plays a key role in quality control and maintenance of stem cell homeostasis (47). Based on the evolution of apoptosis-resistant tumor cells, regulating autophagy may provide a new tumor treatment strategy.

### Autophagy as a Tumor Promoter

Although autophagy inhibits GB progression, it also promotes GB cell survival, proliferation, chemotherapy resistance, and antiapoptotic effects. Studies have shown that rapid proliferation of tumor cells causes metabolic stress, which requires activation of autophagy in order to sustain cell survival. The mechanisms of autophagy promoting GB progression have been largely elucidated.

First, when facing hypoxia or nutrient deprivation, GB cells could produce energy from metabolic substrates by activating autophagy (39). For instance, hypoxia induced by antiangiogenic therapy increases the levels of the autophagy-mediating BCL2-interacting protein 3 (BNIP3) protein, which induces the activation of autophagy in U87 and T98G glioma cell lines as a cytoprotective adaptive response, thereby promoting cell survival. However, these effects can be reversed by addition of the autophagy inhibitor chloroquine (CQ) through the conversion of LC3-I into LC3-II, degradation of sequestosome 1 (SQSTM1/p62), and decrease in BNIP3 expression, leading to a shift from autophagic to apoptotic cell death (48). Moreover, a combination therapy with TMZ and the autophagy inhibitor CQ has been shown to enhance chemotherapy against GB and increase cell apoptosis (49). Second, another important mechanism is through the alteration of autophagy-related proteins like p62, an important autophagy cargo adaptor and autophagy substrate that plays a crucial role in both normal physiology and cancer (50). Some studies have confirmed that overexpression of p62 promotes GB progression by promoting cell proliferation, migration, glycolysis, TMZ resistance, and the nuclear factor kappa B subunit 1 (NF-κB) signaling pathway, and inhibiting autophagy flux and reactive oxygen species (ROS) *in vitro* (50). Third, autophagy confers resistance to chemotherapy and radiotherapy in GB cells. The tumor microenvironment, such as increased ROS level, induces autophagy and inhibits apoptosis by promoting glycolysis to obtain nutritional and energetic requirements. Furthermore, oxidative stress caused by cancer cells also induces autophagy by activating pro-autophagic factors like hypoxia-inducible factor 1 subunit alpha (HIF-1α) and NF-κB (51). Autophagy also facilitates the dissemination of tumor cells and promotes cell invasion and metastasis.

### Phosphoinositide 3-Kinase/Protein Kinase B/Mammalian Target of Rapamycin Pathway Regulates Autophagy

The activated PI3K/AKT/mTOR pathway promotes cell growth and tumorigenesis by inhibiting the activity of autophagy-related proteins, which form the molecular basis of the protective or destructive mechanisms. This is mediated through the regulation of mTOR. Understanding the molecular links between autophagy and the PI3K/AKT/mTOR pathway is the basis for developing more effective therapies.

mTORC1 inhibits autophagy initiation by inhibiting ULK1. Kim et al. reported that mTORC1 inhibited the initial step of autophagy by inactivating, through the phosphorylation of ULK1, a key protein involved in autophagy initiation (52). In fact, ULK1, the uppermost protein of the ULK1–ATG13–FIP200–ATG101 complex, is regulated by the nutrient- and energy-sensitive kinases mTORC1 and AMPK (52, 53). AMPK is activated by liver kinase B1 (LKB1) under nutrient or energy deprivation, and it regulates the activity of mTORC1 by phosphorylating TSC2. Under nutrient abundance, activated mTORC1 inactivates ULK1 by phosphorylation at Ser757 and disrupts the interaction between ULK1 and AMPK, leading to the inhibition of autophagy. Under starvation, AMPK is activated and phosphorylates mTORC1, preventing the phosphorylation of ULK1 by mTORC1, resulting in the initiation of autophagy (52, 53). On the other hand, AMPK directly phosphorylates and activates ULK1 in the cytoplasm, and then ULK1 phosphorylates the transmembrane protein ATG9 and BECN1, thereby enhancing the activity of the autophagy-promoting vacuolar protein sorting 34-kDa (VPS34) complex, followed by recruitment of VPS34 to the phagocytic vesicle, which is necessary for the initiation of autophagy (54). VPS34 is the only class III PI3K in mammals (namely, PIK3C3/VPS34) that phosphorylates phosphatidylinositol to produce PIP3, which is responsible for recruiting downstream components of the autophagy pathway, it is also necessary for autophagosome formation (54). AMPK and mTORC1 may directly regulate VPS34 kinase activity, thereby ensuring the initiation of autophagy in response to cellular stress (55, 56). Other studies also have shown that mTOR regulates the transcription of ULK1 by activating EIF4EBP1, and ribosomal protein S6 kinase (RPS6K) (10). mTOR negatively regulates the stability of ULK1 by phosphorylation of AMBRA1 at Ser52, which induces the polyubiquitination of ULK1 (57). Besides nutrient deficiency, other cellular pressures such as ROS and accumulation and reduction of cell signaling factors also activate autophagy by changing the phosphorylation regulation of ULK1 by AMPK and mTORC1 (58).

Furthermore, mTOR also inhibits the transcription of many autophagy-related proteins, including p62, VPS11, LC3-II, UVRAG, and WIP, through phosphorylation of the transcription factor EB **(**TFEB) at Ser142 and Ser211 (59). TFEB is one of the most important transcription factors that specifically bind to the promoter regions of many genes encoding lysosomal and autophagic proteins, which are involved in lysosomal acidification, cytoplasmic matrix degradation, autophagosome formation, and autophagosome–lysosome fusion (60). TFEB positively regulates the response of autophagosomes and lysosomes to the degradation needs caused by cellular pressures. The transcriptional activity of TFEB is primarily regulated by mTORC1 (59). Under nutrient-rich conditions, mTORC1 phosphorylates TFEB and creates a binding site for the cytoplasmic chaperone 14-3-3, thereby sequestering the inactive TFEB in the cytoplasm (59). However, under nutrient deprivation, TFEB is not phosphorylated by mTORC1 and thus cannot interact with 14-3-3, which allows the translocation of TFEB to the nucleus to induce the transcription of target genes (61, 62).

Recent studies have revealed that mTORC1 phosphorylates UVRAG and inhibits autophagosome maturation in late stages of autophagy (63, 64). UVRAG, which is located in the endoplasmic reticulum (ER) and endosomes, is an important regulator of autophagosome maturation by binding to the homotypic fusion and vacuole protein sorting (HOPS) complex. Besides, UVRAG stimulates the activity of PIK3C3/VPS34 to regulate autophagy (63). Another study revealed that mTORC1 negatively regulates autophagy by modulating the expression of death-associated protein 1 (DAP1), a novel substrate of mTOR that inhibits autophagic flux (65).

EGFR inhibits autophagy by inhibiting microtubule-associated protein chain 3 (MAP1LC3/LC3) and BECN1. LC3, which is the most widely used marker for autophagosomes, plays an indispensable role in autophagosome formation. LC3 expression is associated with a better survival in GB patients with a poor performance score (66). LC3 exists not only as a precursor form (LC3-I) but also as an active form (LC3-II). LC3-I localizes to the cytosol, while LC3-II, after covalent linkage to phosphatidyl ethanolamine (PE), is present on both the cytosolic and intralumenal faces of autophagosomes (67, 68). The LC3 conversion from LC3-I to LC3-II in the cytosol is indicative of the induction of autophagy. Once fused with lysosomes, the intraluminally located LC3-II is degraded by lysosomal hydrolases, and the cytosolically oriented LC3-II is delipidated by ATG4, released from the membrane, and eventually recycled back to LC3-I (69, 70). Another study reported that EGFR expression was negatively correlated with LC3B, and this correlation was most likely a turnover by controlling LC3B protein production, which implies that there is a close interaction between EGFR signaling and autophagy (16). Besides, EGFR amplification inhibits autophagy by maintaining high activation of the Ras/Raf, PI3K/AKT/mTOR and STAT3 signaling pathways, as well as by inhibiting BECN1, which is a key regulator of autophagy (16).

The interplay between the PI3K/AKT/mTOR pathway and the autophagic process is complex. mTOR acts as a central regulator for cell growth and autophagy. Accordingly, based on the fundamental roles of autophagy-related proteins in the regulation of autophagy flux or through regulation by the PI3K/AKT/mTOR pathway, we can target the appropriate proteins for GB prevention and therapy.

### Therapeutic Role of Autophagy in Glioblastoma Treatment Under Phosphoinositide 3-Kinase/Protein Kinase B/Mammalian Target of Rapamycin Pathway Inhibition

There are some evidence indicating that autophagy suppresses the proliferation and migration of cancer cells or enhances the sensitive effects to treatment. For instance, GDC-0941, a highly specific PI3K inhibitor, when combined with TMZ and ionizing radiation (IR), enhances the autophagy response and proapoptotic effects, leading to suppressed cell viability in GB cell lines (9). Mecca et al. reported that PP242 represses GB cell proliferation through induction of high autophagy levels and reduction of cell migration and invasiveness (71). Choi et al. found that treatment with a dual inhibitor of PI3K/mTOR PI-103 increased the cytotoxic and sensitive effects of radiation therapy plus TMZ in GB cells. One of the mechanisms of enhanced radiosensitizing effects is induction of autophagy and apoptosis (72).

The activation of autophagy by PI3K/AKT/mTOR signaling pathway inhibitors is just a process that goes along with cell death (like apoptosis). While PI3K/AKT/mTOR signaling pathway inhibitors clearly induce autophagy, none of the cited studies provides any evidence that they actually promote a pro-death type of autophagy. The pro-death type of autophagy can be defined as: autophagy-mediated cell death, where the induced autophagy triggers apoptosis; and autophagy-dependent cell death, where cell death occurs on the induction of autophagy that is independent of apoptosis or necrosis (73). In many cases, activation of autophagy by the PI3K/AKT/mTOR signaling pathway inhibitors is a pro-survival stress response in GB cells undergoing apoptosis. However, in contrast to the PI3K/AKT/mTOR inhibitors, other established compounds induce autophagic cell death in GB cells by the PI3K/AKT/mTOR signaling pathway inhibition.

Aoki et al. demonstrated that curcumin, a constituent of turmeric, induced autophagy by suppressing the AKT/mTOR pathway, leading to non-apoptotic autophagic cell death in U87 and U373 glioma cells. Interestingly, activation of the AKT pathway inhibited curcumin-induced autophagy and cytotoxicity (8). Maiti et al. reported that treatment with solid lipid curcumin particles (SLCPs) led to the inhibition of the PI3K/AKT/mTOR signaling pathway and increased levels of autophagy, which ultimately resulted in autophagy-related cell death of glioma cells (74). Similarly, Cheng et al. reported that ganoderic acids A (GA-A), the renowned major bioactive triterpenoids from *Ganoderma* mushrooms, had promising cytotoxicity on GB mediated by inhibiting the PI3K/AKT signaling pathway and inducing apoptosis and autophagy. GA-A significantly inhibited the proliferation and migration of GB cells by reducing the expression levels of key proteins involved in the PI3K/AKT/mTOR pathway, including p-AKT, mTOR, and p-P70S6K, and activating autophagy flux by increasing BECN1 and LC3-II while reducing autophagic substrate p62, as well as promoting apoptosis (75). Magnolol and honokiol, extracted from *Magnolia officinalis*, inhibit GB progression by inducing cell cycle arrest and decreasing the expression of p-PI3K, p-AKT, suggesting proliferation inhibition. In addition, the treatment with magnolol and honokiol exerts a synergistic antitumor effect also by inducing autophagy and apoptosis in GB cells (76). Li et al. reported that endothelial-monocyte-activating polypeptide II (EMAP 2), a tumor-derived pro-inflammatory cytokine, promoted autophagic vacuole formation and inhibited the PI3K/AKT/mTOR signaling pathway, thereby inducing autophagy and inhibiting the viability, migration, and angiogenesis of GB (77). The promitotic and adhesion-mediating discoidin domain receptor tyrosine kinase 1 (DDR1) plays a critical role in the modulation of GB therapy resistance. Inhibition of DDR1 combined with radiotherapy and TMZ treatment enhances the sensitivity of the GB treatment and prolongs survival, through a mechanism in which the 14-3-3–BECN1–AKT1 protein complex assembled with DDR1 promotes AKT/mTOR signaling pathways and is crucial for the regulation of autophagy-mediated therapy sensitivity (78).

Although the paradoxical role of autophagy in cancer treatment is still debated, we presume that the induced autophagy by inhibition of the PI3K/AKT/mTOR signaling pathway contributes to suppress cell proliferation, increase sensitivity to the GB treatment, and prolong survival time.

### Autophagy-Mediated Cytoprotection Is Responsible for Resistance to Phosphoinositide 3-Kinase/Protein Kinase B/Mammalian Target of Rapamycin Inhibitor

Primary or recurrent GB treated according to the current standard induces autophagy. However, the enhancement of autophagy by the inhibitors of PI3K/AKT/mTOR signaling leads to tumor cell drug resistance, increased metabolic capacity, and antiapoptotic capacity, which is regarded as a cytoprotective adaptive reaction (15). Therefore, in order to reduce the PI3K/AKT/mTOR pathway inhibitor-induced resistance, the combination of the PI3K/AKT/mTOR pathway and autophagy inhibitors may be an effective strategy to inhibit GB growth and proliferation.

Reduction of the tyrosine kinase activity of EGFR by targeting with TKIs leads to induction of autophagy activity and apoptosis. In contrast, EGFRvIII expression induces resistance to gefitinib and leads to sustained downstream activation of the PI3K/AKT/mTOR pathway (16). More interestingly, the expression of the EGFRvIII variant in GB cells could enhance the activation of autophagy and confer cells with a survival advantage in starvation and hypoxic conditions (16, 79). However, fortunately, autophagy inhibition reduces the resistance to EGFR-targeted drugs (16). In addition, besides being a pan-PI3K inhibitor, PX-866 is also an inhibitor of autophagic flux. In the T98G human glioma cell line, PX-866 simultaneously inhibits PI3K/AKT signaling and TMZ-induced autophagy (cell survival pathway), leading to promotion of apoptosis (80). Accordingly, the combination of autophagy inhibition and traditional cancer therapy provides a theoretical basis for the development of a potential new strategy to treat GB.

Recently, the combination of autophagy inhibitors and PI3K/AKT/mTOR pathway inhibitors has been reported to enhance cell death in various cancers (Table 2). For example, Fan et al. found that combining PI-103, a dual inhibitor of PI3K and mTOR, with 3-methyladenine (3MA) or bafilomycin A1, resulted in significant apoptosis in GB cells with wild-type PTEN (81). Additionally, the clinical dual PI3K/mTOR inhibitor NVP-BEZ235 cooperated with the autophagy inhibitor CQ and led to apoptosis in PTEN-mutant glioma xenografts *in vivo* (81). Li et al. showed that a combination of NVP-BEZ235 and CQ enhanced the inhibitory effect against the growth of human renal cell carcinoma cell line 786-0 cells and induced apoptosis (82). Similarly, the same indications, including enhanced growth inhibition and further apoptosis induction were observed in breast cancer MCF-7 cells treated with PI3K/AKT/mTOR pathway inhibitors in combination with autophagy inhibitors (83). Also, Chang et al. found that treatment of hepatocellular carcinoma cell lines with NVP-BEZ235 combined with the autophagy inhibitor 3MA or ATG5 siRNA resulted in enhanced cell growth inhibition and induction of apoptosis (84). Kim et al. showed that combination therapy with the new PI3K/mTOR dual inhibitor CMG002 and the autophagy inhibitor CQ synergistically increases apoptosis in gastric cancer cells (85). Moreover, SBI-0206965, a specific inhibitor of ULK1/2, suppresses ULK1-mediated phosphorylation. Egan et al. found that SBI-0206965 synergized with three different mTOR inhibitors, including rapamycin, AZD8055, and CQ to induce significantly enhanced apoptotic response in A549 lung cancer cells (86). Furthermore, SAR405, a low molecular mass kinase inhibitor of PIK3C3/VPS34, in combination with mTORC1 inhibitor everolimus produced a significant synergistic effect in reducing the proliferation of renal tumor cells (87).

**Table 2:** Combination therapy with PI3K/AKT/mTOR pathway and autophagy inhibitors for cancer treatment.

**Compound**	**Cancer type**	**Effect**	**References**
PI-103 + 3MA	Glioblastoma	Enhanced apoptosis	(81)
PI-103 + Bafilomycin A1	Glioblastoma	Enhanced apoptosis	(81)
NVP-BEZ235 + CQ	Human renal cell carcinoma	Decreased cell growth and induced apoptosis	(81, 82)
NVP-BEZ235 + 3MA	Hepatocellular carcinoma	Decreased cell growth and induced apoptosis	(84)
CMG002 + CQ	Gastric cancer	Enhanced apoptosis	(85)
SBI0206965 + rapamycin	Lung cancer	Enhanced apoptosis	(86)
SBI0206965 + AZD8055	Lung cancer	Enhanced apoptosis	(86)
SBI0206965 + CQ	Lung cancer	Enhanced apoptosis	(86)
SAR405 + everolimus	Renal tumor	Decreased cell growth	(87)

Based on these findings, we speculate that the functional role of autophagy inhibition in combination with the PI3K/AKT/mTOR pathway inhibition could serve as a mechanism to enhance apoptotic cell death and reduce resistance to therapy, and provide a new insight into the role of autophagy inhibition in the treatment of GB.

## Prospect-Targeting Autophagy in Glioblastoma Therapy

Currently, the standard treatment for GB is surgical resection and radiotherapy as well as adjuvant chemotherapy with TMZ. However, drug resistance to chemotherapy and insensitivity to radiotherapy continue to be a major challenge limiting the efficacy of anticancer drugs. Increasing evidence indicates that while autophagy contributes to the anticancer effects of chemoradiotherapy, it also confers resistance to these therapies (88). Accordingly, the next two sections are about the role of autophagy in chemotherapy and radiotherapy resistance.

### Targeting Autophagy Improves the Efficacy of Chemotherapy

TMZ is the only clinical drug that is currently used in the treatment of GB, but GB patients frequently acquire drug resistance to the therapy. In this section, we take TMZ as an example to discuss the role of autophagy in chemotherapy resistance and introduce the current autophagy inhibitors.

Several mechanisms of TMZ resistance in GB patients have been discerned. The O6-methylguanine-methyltransferase (MGMT), a DNA repair protein, restores the structural integrity of O6-alkylated DNA bases, thereby neutralizing TMZ-induced cytotoxicity (89). In addition, the alterations of other DNA repair pathways like base excision repair (BER) also decrease TMZ sensitivity (90). Currently, the immediate priority is to improve the therapeutic efficacy of TMZ. Autophagy is a promising research direction, as inhibition of autophagy enhances the cell apoptosis and sensitivity of GB cells to TMZ (91). For instance, TMZ usually triggers ER stress-induced autophagy and apoptosis, while the combination of TMZ with an ER stress inhibitor like 4-phenylbutyrate (4-PBA) enhances TMZ-induced cytotoxicity by inhibiting autophagy (92). Moreover, thioridazine, a dopamine antagonist, suppresses autophagy by blocking autophagy at the autophagosome–lysosome junction and impairing fusion between the autophagosomes and the lysosomes, which prevents adaptive metabolic changes associated with TMZ resistance in glioma cell lines. Additionally, thioridazine in combination with TMZ effectively reduces the size of GB tumor xenografts *in vivo*, suggesting the potential role of targeting the autophagy pathway in the treatment of GB (91). It has also been reported that lovastatin could enhance the TMZ efficacy in GB treatment, which may be associated with impaired autophagic flux (93). In summary, the therapeutic efficacy of TMZ can be improved by targeting autophagy in GB, which induces the apoptosis of GB cells. Therefore, the combination of autophagy inhibitors and TMZ is a promising therapeutic strategy for the treatment of GB.

Since autophagy-mediated cytoprotection is responsible for drug resistance, inhibition of autophagy is an effective target for the treatment of GB. The common autophagy inhibitors are described below (Table 1).

Hydroxychloroquine (HCQ) and CQ, as autophagy inhibitors, have shown the best survival benefit in GB patients and significant anti-glioma effect through the inhibition of autophagy by blocking the fusion of autophagosomes with lysosomes (94). Bafilomycin A1 is a specific inhibitor of vacuolar-type H^+^ ATPase (V-ATPase) that inhibits the autophagic flux by preventing the acidification of endosomes and lysosomes. Studies have reported that bafilomycin A1 specifically inhibits the late stages of the autophagy pathway by activating mTOR signaling and disassociating the BECN1–VPS34 complex. In addition, bafilomycin A1 induces the binding of BECN1 to Bcl-2 (apoptosis regulator), which reduces functional autophagy and promotes apoptotic cell death (95). The PI3K inhibitors 3MA (96), wortmannin, and LY294002 have also been used as autophagy inhibitors based on their inhibitory effect on PIK3C3 activity, which is essential for induction of autophagy (37). Lys05 is a newly synthetic lysosomotropic agent that has anti-glioma activity by inducing lysosomal membrane permeabilization and inhibition of autophagy, resulting in decreased cell viability and cell growth, as well as radiosensitivity of glioma U251 and LN229 cells (97).

Besides the autophagy inhibitors, there are some drugs that can be effective by targeting autophagy. Galangin (GG), a flavonoid, induces the formation of autophagic vesicles and protective autophagy in GB cells. A study by Kong et al. revealed that GG combined with CQ for GB treatment enhanced GG-induced apoptosis and pyroptosis, and that was an effective therapeutic strategy for GB (98). N-(4-hydroxyphenyl) retinamide (4-HPR), a retinoid analog, is a potent reagent that has anticancer activity in a variety of tumors, with relatively few adverse side effects *in vivo*. Studies have shown that 4-HPR induces autophagy and apoptosis depending upon its concentration in glioma cells. The inhibition of autophagy at a lower concentration sensitizes high-grade glioma cells to 4-HPR-induced apoptosis, which achieves higher efficacy and prevents the recurrence of high-grade gliomas (99). These studies indicate that the linking between apoptosis and autophagy may contribute to the development of more effective therapies for GB.

However, accumulating evidence has shown that simply using autophagy inhibitors or drugs cannot effectively inhibit cell proliferation in the long term and may even cause cell death at high concentration. Thus, autophagy-inhibitor-based combination therapy should be used for the treatment of GB.

### Targeting Autophagy Contributes to Reduction of Radiotherapy Resistance

Radiotherapy is a highly cost-effective treatment that mainly targets cancer cells by destroying DNA. Radiotherapy frequently induces the recurrence of tumors by activating a variety of signaling cascades, such as the PI3K/AKT/mTOR pathway (100). Moreover, in response to radiotherapy resistance, radiation-induced autophagy is generally considered cytoprotective, which can be used as a target to sensitize cancer cells to radiation. Several lines of evidence have demonstrated that inhibition of autophagy improves the radiosensitivity of tumor cells (101). For example, trifluoperazine (TFP) is a typical antipsychotic drug that inhibits autophagy flux in GB cell lines by increasing the expression of LC3B-II and p62, and disrupting acidification of lysosomes. An additive effect of combining TFP and radiation has been observed, which resulted from TFP-induced impairment of homologous recombination. Additionally, the downregulation of cathepsin L is likely responsible for the radiosensitivity effect of TFP (102). Zheng et al. found that the inhibition of cathepsin D (lysosomal aspartyl protease) by its inhibitor or siRNA attenuated autophagy and enhanced the radiosensitivity in U251 cell lines (103). Taken together, the combination of autophagy inhibitors and radiotherapy showed promising effectiveness, by increasing the sensitivity and enhancing cell apoptosis.

## Discussion

GB is a highly aggressive and recurrent brain tumor for which currently there is no effective treatment. Based on previous studies, the relapse of GB depends on the hyperactivation of the PI3K/AKT/mTOR signaling pathway (22, 104). Autophagy has a dual role in GB therapy with the PI3K/AKT/mTOR inhibitors. On the one hand, autophagy induced by the PI3K/AKT/mTOR inhibitors suppresses GB cell growth. On the other hand, autophagy also promotes GB progression by cytoprotective adaptive response when drug resistance develops, leading to cell growth and survival. Therefore, in order to reduce the resistance caused by the PI3K/AKT/mTOR inhibitors, the use of a combination of autophagy and the PI3K/AKT/mTOR inhibitors may represent a potentially effective strategy for GB therapy. However, in order to develop a more effective treatment, there are many problems that need to be further studied. In this section, we mainly discuss when a combination of autophagy inhibitors is more beneficial for GB therapy, as well as when to regulate autophagy with precise drugs to achieve the effect of tumor adjuvant therapy.

Predicting the therapeutic response of GB to PI3K pathway inhibitors is of great significance and can guide us when to combine with autophagy inhibitors. The traditional methods for evaluating pathway activation include immunohistochemistry (IHC) and Western blotting (WB), which use phospho-specific antibodies to identify pathway components phosphorylated at specific residues (31). The predictive biomarkers, such as p-AKT, p-mTOR, and their downstream substrates are most likely to respond to the PI3K/AKT/mTOR inhibitors. IHC visually shows the intracellular localization of pathway-related proteins, and the localization sites include the plasma membrane, cytoplasm, and nucleus. WB objectively quantifies the expression of pathway-related proteins. The optimal treatment strategy with the PI3K/AKT/mTOR pathway inhibitors can be determined by combining the analysis results of IHC and WB before and after treatment. If autophagy-associated drug resistance develops, the combination of autophagy inhibitors are potential therapeutic agents. However, not all inhibitors of the PI3K/AKT/mTOR pathway can synergize with inhibitors of autophagy. Fan et al. showed that both mTOR and PI3K/mTOR inhibitors can induce autophagy in glioma cells, leading to promoted cell survival (81). Moreover, the combination of inhibitors at different stages of autophagy with PI3K/mTOR inhibitors may produce different effects, which also need to be confirmed by further studies.

Since autophagy plays a crucial role in GB progression, it is important to know precisely when to regulate autophagy to achieve the effect of tumor adjuvant therapy. Tumor development is a complex process, and autophagy has a dual effect on tumor growth at different stages of tumor development (105). On the one hand, autophagy promotes tumor survival by clearing and recycling cellular components to rapidly support cell metabolism and maintain mitochondrial functions under various conditions, including starvation, hypoxia, ER stress, protein folding, and aggregation (36). In addition, over activated protective autophagy, induced by irradiation or chemotherapy, leads to the desensitization of cells to develop resistance. On the other hand, autophagy can also inhibit GB at early tumorigenesis through excessive self-digestion and degradation of essential cellular components like oncogenic protein substrates, leading to cell senescence or cell death (38, 39). The dual role of autophagy in tumorigenesis may be tissue dependent and varies among different stages of tumor growth. Thus, since accurate and individualized treatment is more effective, targeting autophagy needs to be further investigated, and autophagy needs to be studied in different conditions.

## Author Contributions

QX provided useful comments, suggestions, contributed to the conception, and design of the paper. MX draft the paper. LD and QX revised the paper. All authors reviewed and approved the final manuscript.

## Conflict of Interest

The authors declare that the research was conducted in the absence of any commercial or financial relationships that could be construed as a potential conflict of interest.
